# Mechanical Stretching induces the apoptosis of parametrial ligament Fibroblasts via the Actin Cytoskeleton/Nr4a1 signalling pathway

**DOI:** 10.7150/ijms.46354

**Published:** 2020-06-15

**Authors:** Wanling Zeng, Yang Li, Bingshu Li, Cheng Liu, Shasha Hong, Jianming Tang, Li Hong

**Affiliations:** Department of Gynecology and Obstetrics, Renmin Hospital of Wuhan University, Wuhan 430060, Hubei Province, P. R. China.

**Keywords:** Mechanical stretching, Actin cytoskeleton, Apoptosis, Pelvic Organ Prolapse

## Abstract

The anatomical positions of pelvic floor organs are maintained mainly by ligaments and muscles. Long-term excessive mechanical tension stimulation of pelvic floor tissue beyond the endurance of ligaments or muscles will lead to the occurrence of pelvic organ prolapse (POP). In addition, cytoskeletal reconstitution is a key process by which cells respond to mechanical stimulation. The aim of the present study was to investigate the protective effect of actin cytoskeleton to resist mechanical stretching (MS)-induced apoptosis in parametrial ligament fibroblasts (PLFs) and the underlying mechanisms. MS provided by a four‑point bending device could significantly induce apoptosis of PLFs from non-POP patients, which exhibited an apoptosis rate close to that of PLFs from POP patients, and the apoptosis rate was higher following latrunculin A (Lat-A, a potent inhibitor of actin) treatment. In addition, Nr4a1 and Bax expression was increased while Bcl-2 and caspase-3 expression was clearly decreased after treatment with MS and Lat-A. However, the apoptosis induced by MS was reduced when the expression of Nr4a1 was downregulated by siRNA. These outcomes reveal a novel mechanism that links the actin cytoskeleton and apoptosis in PLFs by Nr4a1; this mechanism will provide insight into the clinical diagnosis and treatment of POP.

## Introduction

The female pelvic floor is composed of pelvic floor muscles and pelvic floor connective tissue. During a woman's lifetime, pelvic floor tissues are subjected to various forces, including gravity, pregnancy, childbirth, coughing, defecation, and other forces. The abdominal pressure of healthy adults in the recumbent position is between 5 ~ 7mmHg (1mmHg = 0.133 kpa) and increases during standing, daily activities, physical labor and weight-lifting exercises. When an intermittent or persistent increase in abdominal pressure exceeds the tension produced by contraction of the pelvic floor muscles, symptoms and diseases, such as pain, organ prolapse, and tension urinary incontinence occur 1. Pelvic floor tissues are always affected by mechanical force, which is an environmental factor involved in the survival of pelvic floor cells, but abnormal mechanical force effects are associated with pathological processes in pelvic floor cells. Increased pelvic mechanical pressure, such as that encountered during pregnancy and delivery, is a key risk factor for the onset of pelvic organ prolapse (POP).

The biomechanical properties of tissues and cells have been shown to be abnormal in the pelvic floor-supporting tissue of patients with POP, suggesting that POP is a disease caused by a progressive decline in the biomechanical properties of pelvic floor-supporting tissues 2, but the exact mechanism of POP is unclear. Cytoskeletal reconstitution is a key process by which cells respond to mechanical stimulation 3, 4. The actin cytoskeleton is a dynamic structure composed of filaments that determines the morphology and strength of cells and plays an important role in resisting mechanical tension 5. Most importantly, cytoskeleton recombination facilitates the transmission of extracellular signals into cells, leading to gene transcription and changes in the cell cycle and cell morphology 6. Furthermore, the actin cytoskeleton has been traditionally considered to be a highly dynamic cytoskeletal element at the onset of apoptosis 7. Some important changes in the cytoskeleton are involved in the process of apoptosis and related signal transmission 8. Nuclear receptor subfamily 4, group A, member 1 (Nr4a1) (also known as Nur77) plays roles in regulating growth and apoptosis 9-11. Nr4a1 can induce apoptosis through interacting with Bcl-2^12^. In addition, the close association between Nr4a1 and the actin cytoskeleton plays an important role in long-term memory and neuronal protection 13.

In the present study, we examined the effects of mechanical stretching (MS) on fibroblast apoptosis and investigated the roles of the actin cytoskeleton and Nr4a1 in this process.

## Materials and Methods

### Patients and sample collection

This study followed human subjects and was approved by the Ethics Committee of Renmin Hospital of Wuhan University (Wuhan, China). All patients provided verbal and written informed consent prior to participation in this study. A total of 15 patients were recruited for this study; 8 women who underwent hysterectomy surgery for reasons excluding the presence of malignant tumors and POP served as controls, and 7 patients who underwent hysterectomy surgery for only advanced POP (POP‑Q standard: POP‑Ⅳ) comprised the POP group. All recruited patients had no connective tissue diseases, endometriosis or gynecologic malignancies. Patients who received surgery in the uterosacral ligamental site or had a history of estrogen application within the past three months were excluded from the present study. Tissue specimens (0.5×0.5×0.2 cm^3^) were obtained from part of the parametrial ligaments (the sacral ligament and cardinal ligaments) during surgery.

### Primary cell culture

Samples were obtained from uterosacral ligaments (USLs), and fibroblasts were cultured and purified as described previously 14. Briefly, the USL tissues were cut into pieces (approximately 1 mm^3^), placed in culture bottles and digested with modified collagenase type I (Invitrogen; Thermo Fisher Scientific, Inc., Waltham, MA, USA) and trypsinase (Sigma‑Aldrich, St. Louis, MO, USA). The fibroblasts were grown in serum‑free Dulbecco's modified Eagle's medium (DMEM; HyClone; GE Healthcare Life Sciences, Logan, UT, USA) supplemented with 10% fetal bovine serum (HyClone; GE Healthcare Life Sciences), and 100 U/ml penicillin/streptomycin (Beyotime Institute of Biotechnology, Haimen, China) at 37 °C in an incubator with 5% CO_2_. PLFs were passaged at 85% confluency and were used for subsequent experiments at passages 3-6. For the latrunculin A (Lat-A, a potent inhibitor of actin 15) experiment, PLFs were treated with Lat-A (30 nmol/L) for 24 h.

### Identification of parametrial ligament fibroblasts

The derived cells were characterized by their spindle‑like morphology, and identified by immunohistochemical staining, which indicated positive staining for vimentin and negative staining for cytokeratin and α-SMA, as previously described 14. Cells from passage 3‑6 were cultured in chamber slides to 50% confluence and subsequently washed, fixed, and treated with 0.4% Triton X-100. Endogenous peroxidase activity was blocked with 3% hydrogen peroxide. The PLFs were incubated with mouse anti-human vimentin monoclonal antibody (Santa Cruz Biotechnology, Inc., Dallas, TX, USA; 200 μg/ml) and mouse anti-human cytokeratin monoclonal antibody (Santa Cruz Biotechnology, Inc.; 200 μg/ml) at 4 °C overnight; PBS was used in place of the primary antibody as negative control. After secondary antibody incubation, hematoxylin was used to stain nuclei. Finally, images were collected with a light microscope (Olympus BX53, Tokyo, Japan).

### Mechanical stretching application

A four‑point bending device (SXG4201; Chengdu Miracle Chemicals Co., Ltd., Chengdu, China) was used to exert mechanical strain on the cells 16. In brief, the device was composed of a drive‑control unit, loading unit and strain plates and dishes. The mechanical parameters were set to 2666 με (the loading displacement was 2 mm) and a frequency of 0.1 Hz for 4 h.

### Phalloidin staining

After being rinsed with PBS, fixed with 4% fresh paraformaldehyde for 15 min and treated with 0.4% Triton X-100 for 5 min, the fibroblasts were incubated with FITC-conjugated phalloidin (Enzo Life Sciences, Farmingdale, NY, USA) at 4 °C overnight. DAPI was added to stain cell nuclei. Images were collected by fluorescence microscopy and analyzed by ImageJ v. 1.48 software (NIH, Bethesda, MD, USA).

### Flow cytometry to detect apoptosis

The PLFs in different groups were digested by trypsinase, washed with PBS 3 times and collected. Then 100 μL of binding buffer was used to resuspend the PLFs. Subsequently, 5 μL of PE Annexin V and 5 μL of 7-AAD were added to each group according to the instructions of a PE Annexin V Apoptosis Detection Kit I (BD Biosciences, catalog No. 559763, USA), and the PLFs were gently mixed and incubated at room temperature for 15 min in the dark, following which apoptosis was detected by flow cytometry.

### Small interfering RNA

Primary PLFs were transiently transfected with siRNA (Nr4a1-targeting siRNA) using TransIT®-2020 transfection reagent (Mirus Corp.), according to the manufacturer's instructions. The knockdown efficiency was monitored by assessing changes in the expression of each protein level using Western blot analysis. A non-silencing control siRNA from Cell Signaling was used as a control.

### Immunostaining

Immunocytochemistry was performed as described previously 17. Briefly, PLFs were fixed with 4% fresh paraformaldehyde for 15 min at room temperature and treated with 0.4% Triton X-100 for 5 min, followed by blocking with 5% goat serum for 30 min. Then, the PLFs were incubated with primary antibodies against Nr4a1 (Abcam, 1:200, catalog No. 13851) overnight at 4 °C. The cell nuclei were stained using 4', 6'-diamidino-2-phenylindole (DAPI, ready-to-use, Servicebio, Wuhan, China). Images were collected by fluorescence microscopy and analyzed by ImageJ 1.48r software (NIH, Bethesda, MD, USA).

### Western blot analysis

After exposure to ES or (and) inhibitor, the total proteins of PLFs were prepared using RIPA lysis buffer containing a proteinase inhibitor (BOSTER Biological Technology, Ltd., catalog No. AR0102 and AR1178, Wuhan, China), followed by protein quantification with a bicinchoninic acid assay kit (Beyotime Institute of Biotechnology, catalog No. P0010, Haimen, China). A total of 20 μg of total cellular protein was mixed with gel loading buffer, separated by 10% SDS-PAGE and transferred onto polyvinylidene fluoride membranes (PVDF, Merck KGaA). Membranes were blocked with 5% nonfat milk-TBST (TBS with 0.1% Tween 20) for 1 h at room temperature and washed with TBST twice, followed by incubation with the following rabbit primary antibodies purchased from Abcam: anti-Bcl-2 (1:1,000, catalog No. ab32124), anti-Bax (1:8000, catalog no. ab53154), anti-caspase 3 (1:500; catalog No. ab13847) and anti-GAPDH (1:2,000, catalog No. ab9485, served as an internal reference control) overnight at 4˚C. Then, the membranes were incubated with fluorescence-labeled secondary antibodies (1:10,000; IRDye700 and IRDye800, goat anti-rabbit, catalog No. 926-32211, LI-COR Biosciences, Lincoln, NE*,* USA) for 1 h at 37 °C after washing. The reactive bands were detected with an Odyssey^®^ infrared imaging system (LI-COR Biosciences, Lincoln, NE*,* USA). The band densities for each sample were normalized against the density of GAPDH, and data were obtained from three experiments.

### Reverse transcription-quantitative polymerase chain reaction (RT-qPCR)

The mRNA expression levels of Nr4a1, Bcl2, Bax, and Caspase 3 in PLFs were evaluated by RT-qPCR. The primers used for amplification were purchased from Beijing SBS Genetech Co., Ltd. (Beijing, China) (Table 1). The total RNA was extracted from PLFs using TRIzol^®^ reagent (Invitrogen; Thermo Fisher Scientific, Inc., Waltham, MA, USA) following the manufacturer's protocol. cDNA was prepared by the reverse transcription of total RNA(100 ng) using a Revert Aid First Strand cDNA Synthesis kit (catalog No. k1622; Thermo Fisher Scientific, Inc. USA) and reaction mixture aliquots (1 μL) were used as templates for PCR. qPCR was performed on an Applied Biosystems 7500 Real-Time system (Applied Biosystems, Thermo Fisher Scientific, Inc. USA) using SYBR^®^ Premix Ex Taq^™^ reagent (catalog No. DRR041; TakaRa Bio, Inc., Otsu, Japan). Target gene mRNA expression levels were normalized to the expression of the housekeeping gene GAPDH for quantification. Each sample was analyzed in triplicate to ensure accuracy.

### Statistical analysis

Data are presented as the mean ± standard deviation for each group and were analyzed by one-way analysis of variance using GraphPad Prism 5.0 (GraphPad Prism INC, CA, USA). Multiple means were compared by Tukey's test. Differences of *p*<0.05 were considered statistically significant.

## Results

### Cell identification

**Figure 1:**
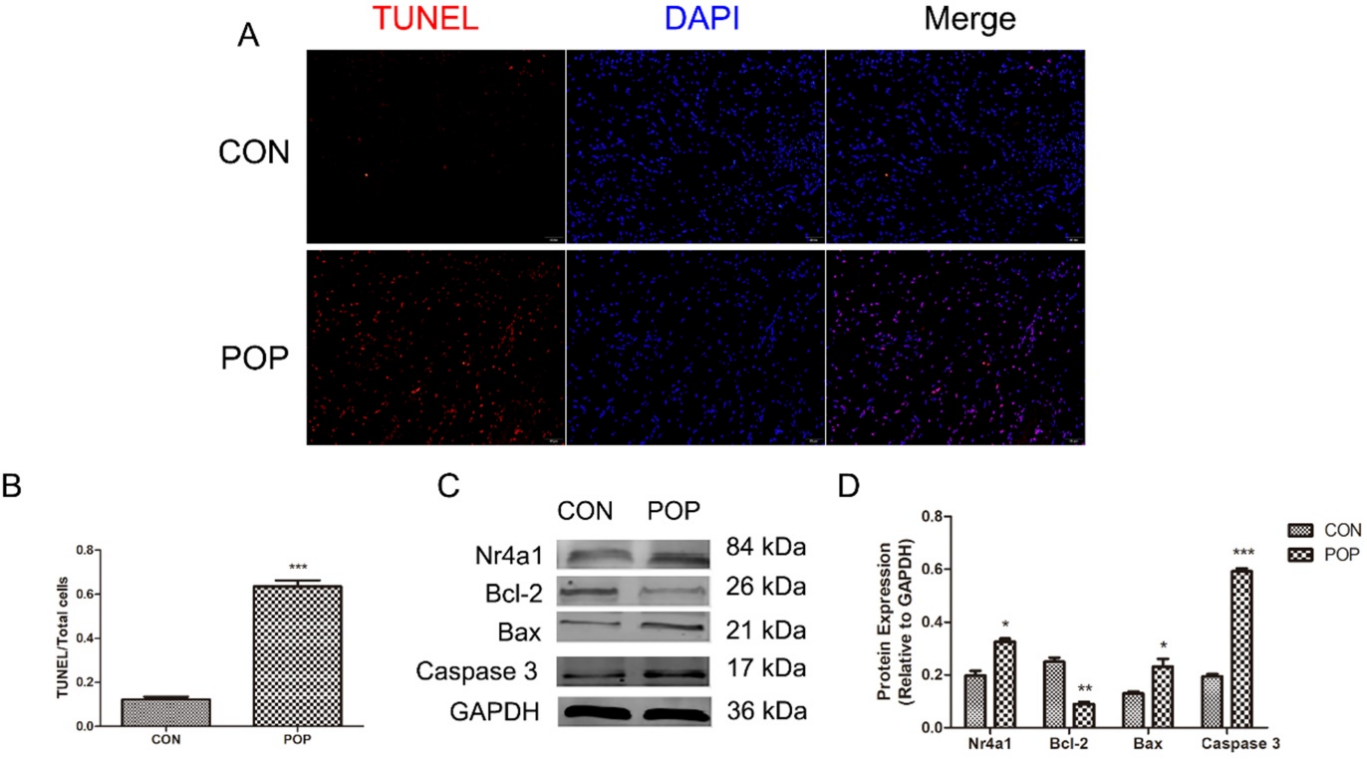
Identification of primary parametrial ligament fibroblasts (PLFs). Immunofluorescence staining for vimentin (A), cytokeratin (B) in cultured PLFs and a merged image (C). (D), Primary cultured PLFs visualized by light microscopy (magnification: 200×).

The fibroblasts isolated from the uterosacral ligaments exhibited stellate, bipolar, and spindle like shapes under an inverted microscope. Immunostaining of the cells isolated from the two groups revealed the strong cytoplasmic expression of vimentin and negative staining for cytokeratin (Figure 1). These outcomes confirmed the origin of the connective tissue fibroblasts.

### Increased apoptosis in the uterine sacral ligament tissue of patients with POP

**Figure 2:**
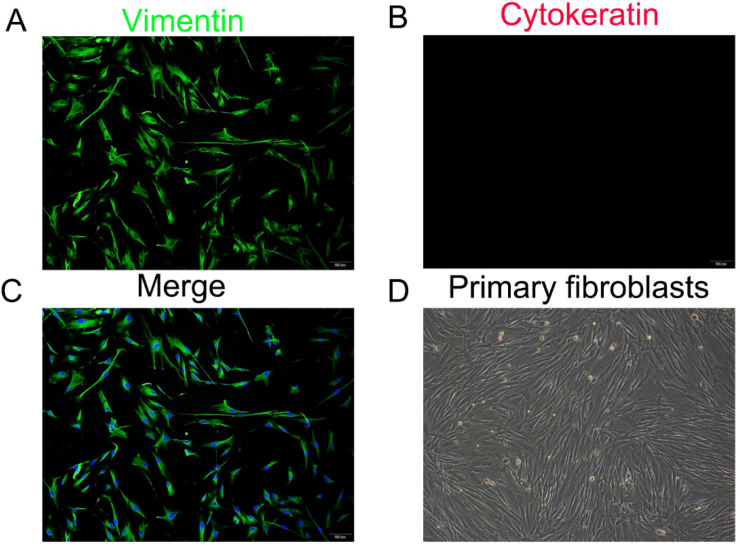
Detection of apoptosis of the uterine sacral ligament. A, TUNEL staining of the uterine sacral ligament, magnification: 200×. B, The ratio of TUNEL-positive cells to the total number of cells. C and D, Protein levels in the uterine sacral ligament of POP and control groups were determined by Western blotting and normalized to those of GAPDH. * indicates p < 0.05, ** indicates p < 0.01, *** indicates p < 0.001. (CON: the uterine sacral ligament obtained from patients without POP; POP: the uterine sacral ligament obtained from patients with POP).

We observed a higher apoptosis rate in the uterine sacral ligament tissue of women with POP compared with women in the control group (Figure 2A, B). Of note, apoptosis-related protein levels were also significantly different, and the expression of Bax and Caspase 3 was increased in the POP group. In contrast, the level of Bcl-2 was significantly decreased in the POP group compared to the control group (Figure 2C, D). Furthermore, Nr4a1 expression showed the same changes as apoptosis (Figure 2C, D). The above data suggested that the apoptosis of uterine sacral ligament cells was increased in POP patients and that Nr4a1 is involved in the pathogenesis of POP.

### The status of cell apoptosis in the uterine sacral ligament

We observed that the uterine sacral ligament tissues of the POP patients presented an increased rate of cell apoptosis compared to that of the control group (Figure 2A, B). Furthermore, the uterine sacral ligament tissues from POP patients more highly expressed Bax and Caspase 3 but not Bcl-2 (Figure 2C, D). It may be that the pelvic floor ligament tissues of patients with POP have been exposed to abnormal mechanical stimulation for a long time, which has affected the normal metabolism of the cells in the uterine sacral ligament tissues. Interestingly, the expression of Nr4a1 was also higher in the POP group than in the control group (Figure 2C, D), which suggested that Nr4a1 plays a role in the occurrence of POP.

### Mechanical stretching induces the apoptosis of parametrial ligament fibroblasts

**Figure 3:**
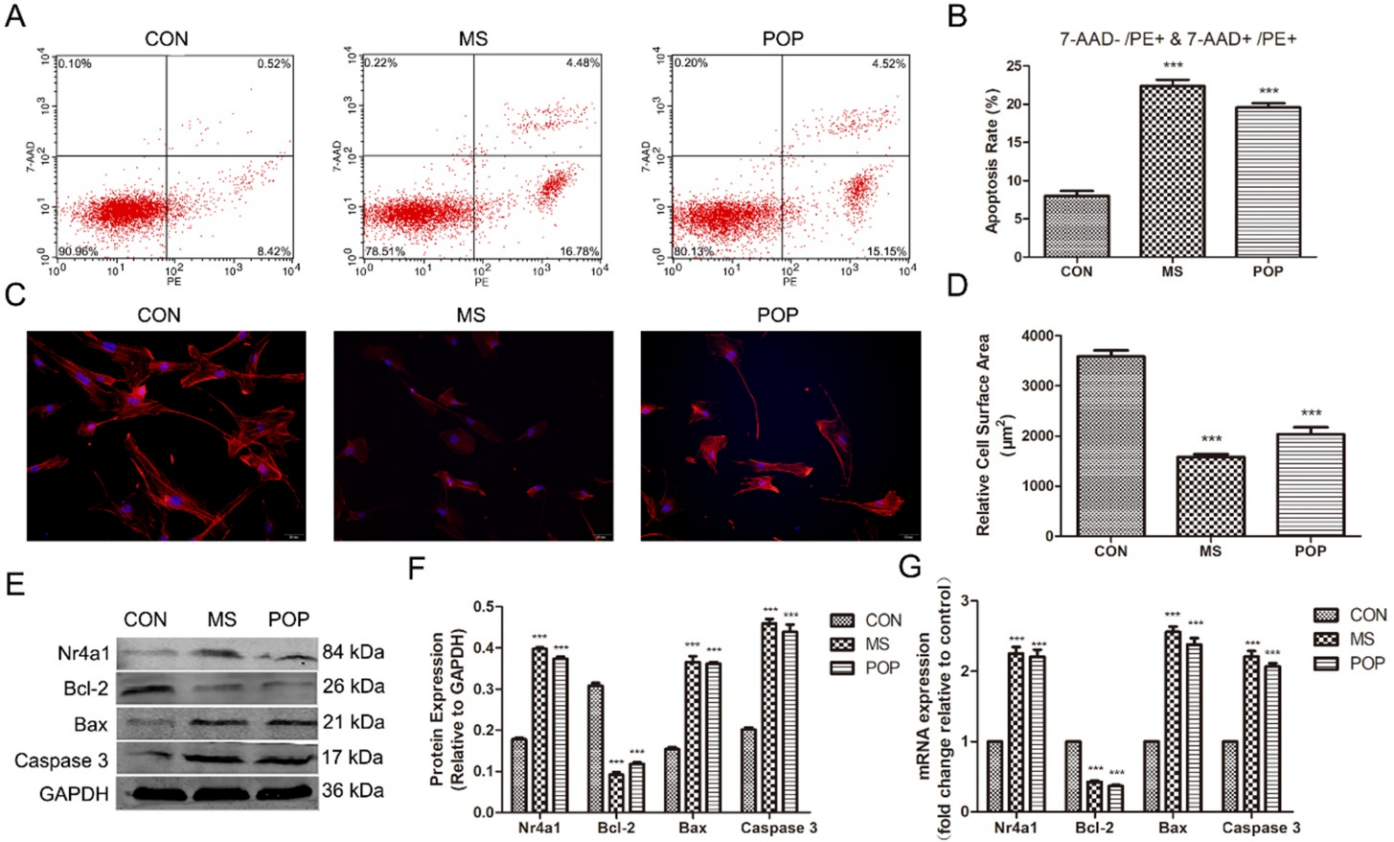
Mechanical stretching increased the apoptosis rate and actin cytoskeleton disassembly in PLFs. A, Cell apoptosis was assessed by flow cytometry analysis. The apoptosis rate was determined as the percentage of PE-positive cells, with early apoptotic cells negative for 7-AAD and late apoptotic cells positive for 7-AAD. B, Quantified apoptosis rates in each group. C, PLFs were stained with phalloidin and imaged by fluorescence microscopy. Red fluorescence delineates the cell cytoplasm; blue fluorescence delineates nuclei (magnification: 200×). D, The relative cell surface areas were quantified by ImageJ software. E, Protein levels in PLFs were determined by Western blotting and normalized to those of GAPDH. F, Band intensities were quantified by Quantity One. G, mRNA levels in PLFs were quantified by real-time RT-PCR and normalized to those of GAPDH. *** indicates p < 0.001. (CON: PLFs isolated from patients without POP; MS: PLFs isolated from patients without POP that were exposed to mechanical stretching; POP: PLFs isolated from patients with POP).

To investigate the effects of abnormal mechanical force on fibroblasts, we established a cellular mechanical stretching loading model. As expected, mechanical stretching induced a potent increase in apoptosis (early apoptosis: 7-AAD-/PE+ and late apoptosis: 7-AAD+/PE+) in the MS group (Figure 3A, B). Moreover, we discovered that the levels of Bax and Caspase 3 were significantly increased, while those of Bcl-2 were decreased after exposure to MS. Notably, MS promoted the expression of Nr4a1 (Figure 3E-G). Importantly, MS also suppressed the expression of F-actin, which indirectly indicates the depolymerization of actin cytoskeleton (Figure 3C, D). Interestingly, similar differences in the apoptosis rate, disassembly of the actin cytoskeleton and expression of related proteins between the PLFs from POP patients and the PLFs from controls after loading MS were observed (Figure 3A-G). This suggests that mechanical force is involved in the occurrence and development of POP, which induces PLF apoptosis and disassembly of the actin cytoskeleton.

### Disassembly of the actin cytoskeleton promotes MS to induce the apoptosis of PLFs

**Figure 4:**
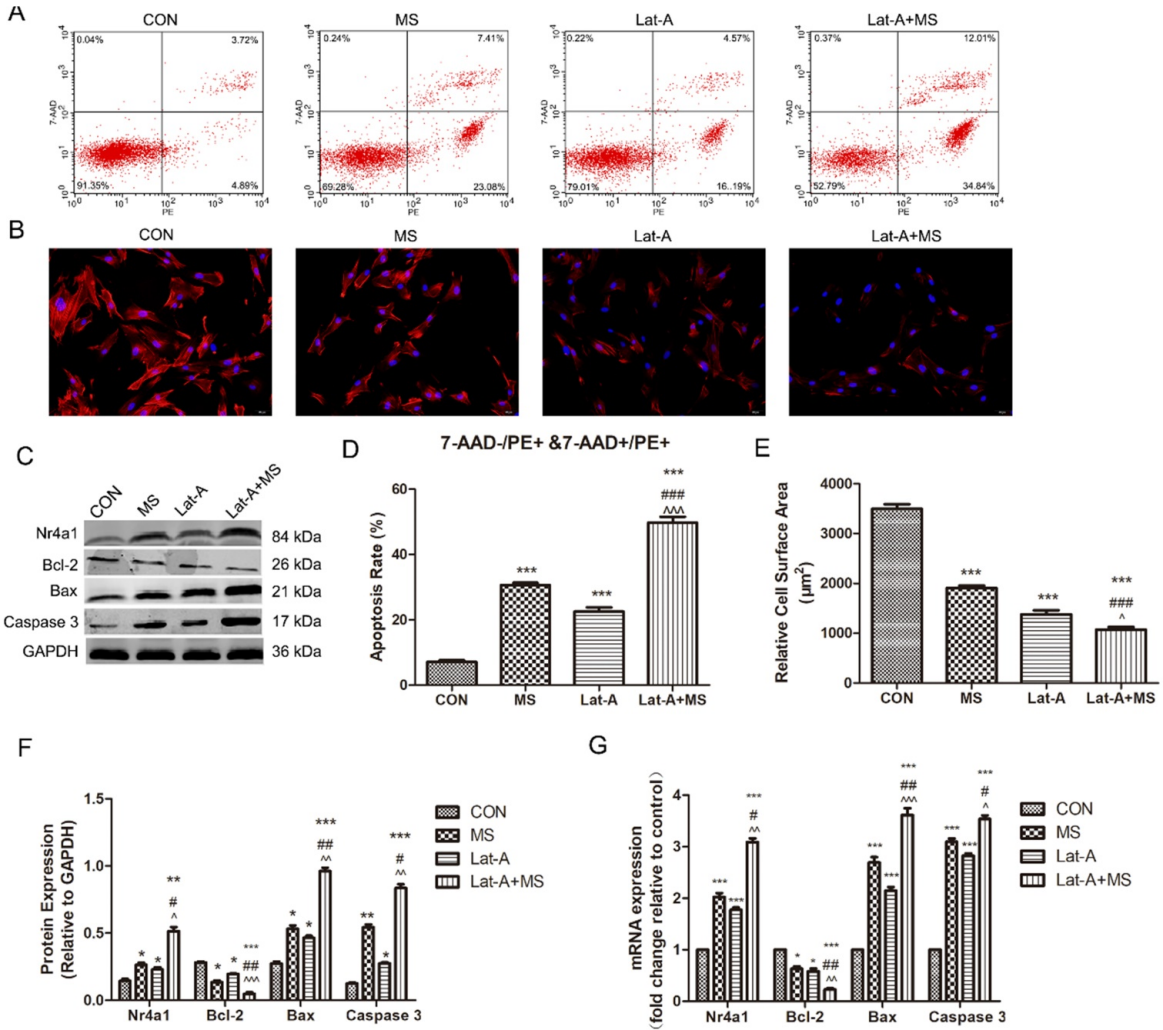
The effect of mechanical stretching on apoptosis after actin cytoskeleton disassembly. A, Cell apoptosis was detected by flow cytometry analysis; B, Quantified apoptosis rates in each group. C, PLFs were stained with phalloidin and imaged by fluorescence microscopy (magnification: 200×). D, Relative cell surface areas were quantified by ImageJ software. E, Protein levels in PLFs were determined by Western blot analysis and normalized to those of GAPDH. F, Band intensities were quantified by Quantity One. G, mRNA levels in PLFs were quantified by real-time RT-PCR and normalized to those of GAPDH. * indicates p < 0.05, ** indicates p < 0.01 and *** indicates p < 0.001 compared with the CON group; # indicates p < 0.05, ## indicates p < 0.01 compared with the MS group; ^ indicates p < 0.05, ^^ indicates p < 0.01 and ^^^ indicates p < 0.001 compared with the Lat-A group. (CON: PLFs isolated from patients without POP; MS: PLFs isolated from patients without POP and exposed to mechanical stretching; Lat-A: PLFs isolated from patients without POP and exposed to Lat-A; Lat-A+MS: PLFs isolated from patients without POP and exposed to Lat-A and mechanical stretching).

We then sought to identify binding partners involved in mechanical stretching-induced apoptosis. Lat-A, which sequesters monomeric actin, resulting in the rapid depolymerization of actin microfibrils^18^, was used to disrupt the actin cytoskeleton. Lat-A induced a dramatic increase in the apoptosis rate of PLFs and induced disassembly of the actin cytoskeleton (Figure 4A-E). In addition, increased disassembly of the actin cytoskeleton enhanced the expression of Nr4a1, Bax and Caspase 3 and decreased the expression of Bcl-2 (Figure 4C, F, G). Furthermore, MS had a stronger effect on apoptosis induction than stimulation with MS or Lat-A along (Figure 4A-G), suggesting that disassembly of the actin cytoskeleton has potent effects on the MS-induced apoptosis of PLFs and that Nr4a1 is regulated by the actin cytoskeleton.

### Apoptosis of PLFs induced by MS via actin cytoskeleton is suppressed by Nr4a1 silencing

**Figure 5:**
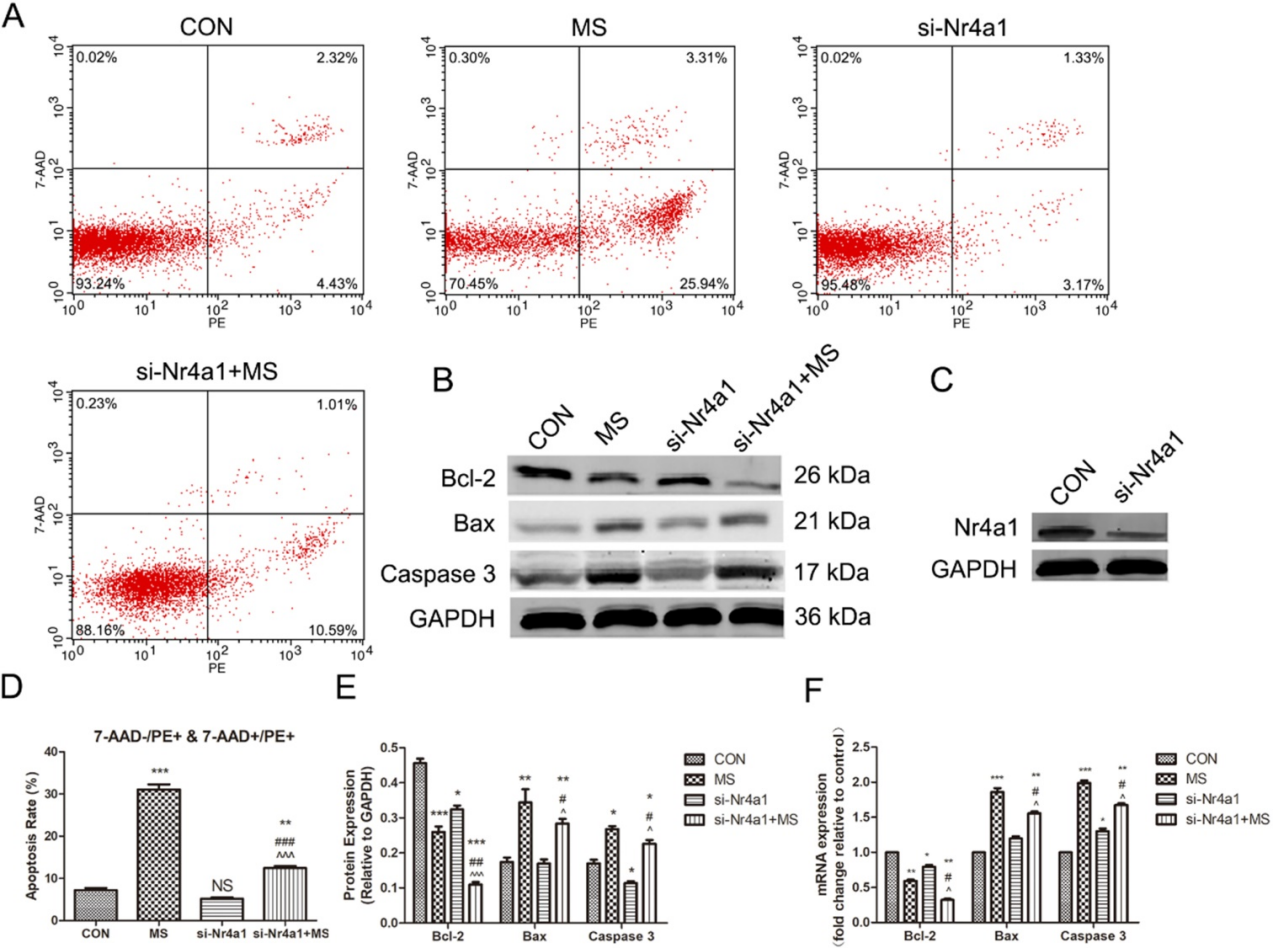
The effect of mechanical stretching on apoptosis after Nr4a1 deficiency. A, Cell apoptosis was detected by flow cytometry analysis. B, Protein levels in PLFs were determined by Western blot analysis and normalized to those of GAPDH. C, The levels of Nr4a1 after Nr4a1 gene interference. D, Quantified apoptosis rates in each group. E, Band intensities were quantified by Quantity One. F, mRNA levels in PLFs were quantified by real-time RT-PCR and normalized to those of GAPDH; * indicates p < 0.05, ** indicates p < 0.01 and *** indicates p < 0.001 compared with the CON group; # indicates p < 0.05, ## indicates p < 0.01 compared with the MS group; ^ indicates p < 0.05 ^^^ indicates p < 0.001 compared with the si-Nr4a1 group. (CON: PLFs isolated from patients without POP; MS: PLFs isolated from patients without POP and exposed to mechanical stretching; si-Nr4a1: si-Nr4a1-mediated transfection was used to silence Nr4a1 in PLFs; si-Nr4a1+MS: si-Nr4a1-treated cells treated with mechanical stretching).

We next investigated the functional impact of Nr4a1 silencing on MS-induced apoptosis to illustrate the importance of Nr4a1 in this process. Nr4a1 silencing alone did not reduce apoptosis (Figure 5A, D), but could suppress the expression of Bcl-2 and Caspase 3 (Figure 5B, E, F), which was also observed by Lin 12. However, in contrast to the effects of Lat-A, Nr4a1 silencing reversed the pro-apoptotic effect of MS (Figure 5 A, B, D-F). The above data illustrate that Nr4a1 may interact with Bcl-2 to participate in MS-induced apoptosis.

## Discussion

The molecular mechanisms of POP remain unclear. Increasing age as well as multiparity are the main factors thought to be involved in POP. The pelvic floor can be thought of as a biomechanical structure due to the complex interaction between the vagina and its supportive structures, which are designed to withstand downward descent of the pelvic organs in response to increased abdominal pressure. However, POP occurs when the pelvic supportive tissues are subjected to abnormal mechanical stimulation 2. The complete tensile tissue structure consists of intracellular matrix and extracellular matrix (ECM), of which extracellular collagen and elastin are the main stress components, and intracellular mechanical resistance is maintained by the cytoskeleton 19. Furthermore, fibroblasts, which are not only the primary ECM-secreting cells but also the main components of ligament tissue, maintain the anatomical positions of the pelvic organs by resisting multiple forces. Therefore, in this study, we investigated changes in PLFs under mechanical stretching.

Abnormal mechanical stimulation damages connective tissues and cells and cause changes in the biological properties of fibroblasts 16, 20. In this study, we observed similar apoptotic outcomes in the PLFs of POP patients and non-POP patients with MS. We simulated mechanical stimulation of the pelvic floor connective tissue and established a mechanical model of fibroblasts, which was widely applied in our previous study 16, 20 and another study 21. After mechanical stretching exposure, PLFs from non-POP patients showed an increased apoptosis rate and disassembly of the actin cytoskeleton. Furthermore, the apoptosis of PLFs was more severe when the actin cytoskeleton was destroyed prior to mechanical stretching. It is likely that stretching conditions, and disassembly of the actin cytoskeleton, induce the apoptosis of PLFs. Therefore, we speculate that mechanical stretching induces the apoptosis of PLFs by destroying actin cytoskeleton, which is similar to the notions that the actin cytoskeleton regulates apoptosis 22 and that mechanical stretching induces actin cytoskeleton remodeling 23.

Furthermore, in this study, the protein level of Nr4a1, an important regulator of apoptosis, was increased in the parametrial ligament tissue of POP patients and after disassembly of actin cytoskeleton by Lat-A. When the actin cytoskeleton was damaged by mechanical stretching, the expression of Nr4a1 was also increased, and upon exposure to Lat-A, it was even more remarkably increased. So, we speculate that actin cytoskeleton, located in the upstream of the pathway, regulates the expression of Nr4a1, which form the actin cytoskeleton /Nr4a1 signaling pathway. Furthermore, changes in the apoptosis of PLFs were consistent with changes in Nr4a1 expression. This may suggest that mechanical stretching can induce disassembly of the actin cytoskeleton, then increase the level of Nr4a1 and regulate cell apoptosis. Therefore, we aimed to investigate whether the interference of Nr4a1expression would affect the apoptosis of PLFs induced by mechanical stretching. As expected, MS-induced apoptosis was reduced by Nr4a1 knockdown. A large number of studies have shown that Nr4a1 can regulate apoptosis 24, 25. In addition, Chen Y et al. 13 reported that Nr4a1 mediates transcriptional regulation of the actin cytoskeleton. In this study, we have proposed for the first time that the actin cytoskeleton induces apoptosis through regulating the activation of Nr4a1.

In conclusion, our results reveal an important pathogenic mechanism of POP in which mechanical stretching can induce actin cytoskeletal depolymerization, and then promote apoptosis via Nr4a1. This study presents a novel perspective on the onset of POP and may provide new insights and strategies for the treatment and prevention of POP. However, further research is needed to confirm the underlying mechanism by which disassembly of the actin cytoskeleton regulates the activation of Nr4a1 in the process of mechanical force-induced POP.

## Figures and Tables

**Table 1:** The primer sequences used for RT-qPCR

Gene	Gene no.	Forward primer (5'-3')	Reverse primer (5'-3')
GAPDH	NM_002046.6	GCACCGTCAAGGCTGAGAAC	TGGTGAAGACGCCAGTGGA
Nr4a1	NM_001202233.1	ACCCACTTCTCCACACCTTG	ACTTGGCGTTTTTCTGCACT
Bcl-2	NM_00633.2	TGGCCAGGGTCAGAGTTAAA	TGGCCTCTCTTGCGGAGTA
Bax	NM_001291428.1	TTGCTTCAGGGTTTCATCCA	AGACACTCGCTCAGCTTCTTG
Caspase 3	NM_004346.4	AGGTATCCATGGAGAACACTG	ACCAGACCGAGATGTCATTCC

## Abbreviations

POP: pelvic organ prolapse
PLFs: parametrial ligament fibroblasts
MS: mechanical stretching
Nr4a1: Nuclear receptor subfamily 4, group A, member 1
ECM: extracellular matrix
